# A comprehensive investigation of *Clerodendrum Infortunatum* Linn. using LC-QTOF-MS/MS metabolomics as a promising anti-alzheimer candidate

**DOI:** 10.1038/s41598-024-82265-2

**Published:** 2025-01-05

**Authors:** Fatma Atef, Mostafa A. Abdelkawy, Basma M. Eltanany, Laura Pont, Ahmed M. Fayez, Mohamed F. Abdelhameed, Fernando Benavente, Inas Y. Younis, Asmaa M. Otify

**Affiliations:** 1 Boulaq El-dakrour general hospital, Giza, 12617 Egypt; 2 Department of Pharmacognosy, Faculty of Pharmacy, Cairo University, Cairo, 11562 Egypt; 3 Department of Pharmaceutical Analytical Chemistry, Faculty of Pharmacy, Cairo University, Cairo, 11562 Egypt; 4 Department of Chemical Engineering and Analytical Chemistry, Institute for Research on Nutrition and Food Safety (INSA·UB), University of Barcelona, 08028 Barcelona, Spain; 5 Serra Húnter Program, Generalitat de Catalunya, 08007 Barcelona, Spain; 6 Department of Pharmacology, Faculty of Pharmacy, Hertfordshire University, Cairo, 11835 Egypt; 7 Pharmacology Department, National Research Centre, Dokki, 12622 Cairo Egypt

**Keywords:** Alzheimer’s disease, Anticholinesterase, *Clerodendrum*, LC-MS/MS, Metabolomics, Neurotransmitters, Mass spectrometry, Biochemistry

## Abstract

Alzheimer’s disease (AD) poses a global health challenge, demanding innovative approaches for effective treatments. *Clerodendrum infortunatum* Linn. (Lamiaceae) is a shrub traditionally used as a medicinal plant to treat inflammation, skin diseases, and bronchitis. This study aims to identify the main bioactive metabolites in *C. infortunatum* using LC-QTOF-MS/MS and investigate its potential in protecting against cognitive decline in rats with scopolamine-induced AD disease. Metabolite profiling was performed on the methanol extract of the plant’s aerial parts using LC-QTOF-MS/MS. The inhibitory activity of the acetylcholinesterase enzyme was measured *in vitro.* To evaluate the cognitive effects, the methanol extract was orally administered at three doses (100, 200, and 400 mg/kg) to scopolamine-induced AD rats, and their cognitive functions were assessed using the novel object recognition test. Additionally, acetylcholinesterase enzyme activity, as well as the levels of acetylcholine, dopamine, noradrenaline, glutathione, malondialdehyde, tumor necrosis factor-α, interleukin-1β, and amyloid-β in the rat hippocampus, were measured using ELISA, followed by histopathological evaluation. A total of 79 metabolites, spanning various chemical classes, such as organic acids, phenolic acids, phenylpropanoids and phenylethanoids, flavonoids, coumarins, other phenolics, and fatty acids and their derivatives, were identified. The results showed that the extract promoted enhanced cognitive functions in the novel object recognition test. Scopolamine administration significantly altered the acetylcholinesterase enzyme activity and biomarker levels in the rat’s hippocampus. However, treatment with *C. infortunatum* at 200 and 400 mg/kg almost restored these neurotransmitter levels to normal, which was further confirmed by histopathological analysis. This study demonstrates the therapeutic potential of *C. infortunatum* in mitigating cognitive decline in AD, with its first metabolite profiling revealing a range of bioactive compounds. The extract improved cognitive function in scopolamine-induced AD rats, restored acetylcholinesterase activity, normalized neurotransmitter levels, and reduced oxidative stress and inflammation. These findings suggest that *C. infortunatum* is a promising candidate for the development of natural therapies targeting AD.

**Supplementary Information:**

The online version contains supplementary material available at 10.1038/s41598-024-82265-2.

## Introduction

Alzheimer’s disease (AD) has become one of the most significant global health concerns of this century, affecting millions of individuals worldwide. According to recent predictions, the number of people with elderly dementia related to AD is expected to reach around 152 million by 2050^1^. AD is a neurological disorder characterized by memory loss and deterioration of several mental faculties^2^. Individuals suffering from AD commonly exhibit neurofibrillary tangles, amyloid plaques of amyloid-β (Aβ) protein, and a reduction in neuronal synapses within the brain^2^. Moreover, age-related neurodegeneration and cognitive decline are believed to be significantly influenced by oxidative stress, which is defined as an imbalance in the generation of radical reactive oxygen species (ROS) and antioxidative defense. One of the early events in AD is neuro-inflammation, which likely precedes the manifestation of Aβ deposits^3^. The intricate interplay among these factors contributes to the observed deterioration in both cognitive and motor abilities^4^. The main trigger of AD is dysfunction in the cholinergic system, which includes malfunctioning neurotransmitters, receptors, and cholinergic neurons, resulting in diminished cognitive function^5^. Consequently, elevating acetylcholine (ACh) levels stands as a key objective in AD treatment. This is accomplished through the inhibition of the acetylcholine esterase (AChE) enzyme^6^. Presently, symptomatic treatments for AD, such as rivastigmine, galantamine, and donepezil (DON), predominantly target memory enhancement by inhibiting the AChE enzyme. However, these drugs are often associated with undesirable side effects^4^. Additionally, AD is a multifaceted condition with multiple contributing factors, rendering the “one change, one disease, one drug” approach ineffective^7^. Therefore, there exists a pressing need for novel, natural medications that can decelerate or halt AD progression while minimizing the side effects commonly associated with these synthetic drugs.

Plant-based remedies have shown promise in effectively slowing down the progression and alleviating symptoms of AD. Particularly interesting are plants such as *Curcuma longa*, *Centella asiatica*, *Ginkgo biloba*, *Zingiber officinale*, *Allium sativum*, and *Clerodendrum infortunatum* (*C. infortunatum*), which present potential antioxidant, anti-inflammatory, anticholinesterase, and anti-amyloidogenic properties^7,8^. *C. infortunatum*, a shrub in the Lamiaceae family commonly found in India, possesses various medicinal properties. It has been reported to exhibit free radical scavenging and wound healing effects^9^. Since some research suggests a connection between Aβ protein toxicity and an increase in ROS, these antioxidant properties may be essential in combating oxidative stress, a critical trigger in AD^10^. However, there is limited research on *C. infortunatum* and its association with neurological deterioration, but some authors have demonstrated promising memory enhancing effects^11^. Additionally, there is limited phytochemical data available regarding the complete profile of *C. infortunatum* constituents, including potential bioactive metabolites. Presently, mass spectrometers with quadrupole time-of-flight (QTOF) mass analyzers are widely utilized for the untargeted metabolite profiling of natural products as they can acquire MS and tandem MS (MS/MS) spectra with excellent mass accuracy and resolution. Accordingly, recent studies have shown the successful application of liquid chromatography-quadrupole time-of-flight tandem mass spectrometry (LC-QTOF-MS/MS) for the global metabolite profiling of constituents in medicinal and edible plants^12^.

In this study, untargeted LC-QTOF-MS/MS metabolomics was conducted to profile the methanol extract of the aerial parts of *C. infortunatum*. Subsequently, the anti-AD potential of the methanol extract was investigated in vitro on the AChE enzyme and in vivo in scopolamine (SCOP)-induced AD rats. SCOP was administered to induce AD in rats, as it is known to increase AChE enzyme activity and ROS, accumulate Aβ protein, and disrupt neurotransmitters^10^. The rats underwent evaluation with the novel object recognition (NOR) test to evaluate their memory and cognitive abilities. Additionally, AChE enzyme activity and various biomarker levels were measured in the rat hippocampus, including neurotransmitters (ACh, noradrenaline (NA), and dopamine (DA)), anti-oxidant parameters (glutathione (GSH) and malondialdehyde (MDA), anti-inflammatory parameters (tissue necrosis factor-α (TNF-α) and interleukin-1β (IL-1β)), and an AD marker, Aβ protein. Finally, the rat brains were subjected to histopathological assessment. To the best of our knowledge, this is the first comprehensive LC-QTOF-MS/MS analysis conducted on *C. infortunatum* aerial parts. The proposed integrated approach unveils the anti-AD potential of *C. infortunatum* and identifies the metabolites responsible for such functionality. These findings hold promise for the future development of novel dietary supplements, nutraceuticals, or drugs for the amelioration and prevention of AD.

## Materials and methods

### Plant material and preparation of plant extracts

The aerial parts of *C. infortunatum* were collected in March 2021 from Mazhar Botanical Garden, Giza, Egypt, and authenticated by Agriculture Engineer Therese Labib, a plant taxonomy consultant of the Ministry of Agriculture (Giza, Egypt). The plant material was collected with permission in compliance with national guidelines from the Agriculture Research Center, Giza, Egypt at “9 Cairo University Road, Giza District, Giza Governorate”. Samples of the plant material were deposited in the herbarium of the Faculty of Pharmacy, Cairo University, Cairo, Egypt (sample No.10.3.2021). Following air-drying, the aerial parts were ground into powder using a powder grinder. Dried powder (5 kg) was repetitively macerated with a methanol-water mixture (85:15, v/v) until exhaustion (25 L) at room temperature. All extracts were filtered using Whatman No. 1 filter paper after maceration. The solvent was evaporated in a rotary evaporator at 45 ºC (BUCHI Rotavapor R-300, Cole-Parmer, Vernon Hills, USA), resulting in 500 g of total extract, which was stored at 4 ºC. For In vitro analysis, 10 mg of the extract was dissolved in 1 mL of dimethyl sulfoxide (DMSO). The resulting DMSO stock solution was then diluted to the required concentrations, maintaining a final DMSO concentration of less than 0.1% throughout the experiment to minimize any potential solvent effects on the extract’s bioactivity. The use of DMSO effectively dissolves plant extracts that may not completely dissolve in water or other solvents. Three biological replicates were prepared and extracted in parallel under the same conditions.

### Drugs and chemicals

Methanol was purchased from Piochem Company for Pharmaceuticals and Chemicals (Cairo, Egypt). SCOP and DON were provided by Merck (Darmstadt, Germany) and Pfizer (Cairo, Egypt), respectively, and both were prepared in saline solution. Formalin, DMSO, paraffin, xylene, eosin, and hematoxylin stains were purchased from Sigma-Aldrich (Burlington, USA). Formic acid (≥ 95.0%), acetonitrile, water, and methanol (LC-MS grade) were supplied by Merck (Darmstadt, Germany).

### LC-QTOF-MS/MS metabolite profiling

For LC-QTOF-MS/MS analysis, the samples were prepared by dissolving 10 mg of the dried *C. infortunatum* methanol extract in 1 mL of methanol, assisted by sonication. The resulting solutions were centrifuged at 13,000 x g for 10 min, and the supernatants were filtered through a 0.22 μm syringe nylon filter. The LC-QTOF-MS/MS analyses were carried out using a 1260 Infinity liquid chromatograph coupled to a 6546 LC/QTOF mass spectrometer with an orthogonal electrospray ionization (ESI) interface (Agilent Technologies, Waldbronn, Germany). Metabolite profiling in both negative and positive ESI mode involved injecting 5 µL of sample into a Zorbax SB-C18 (5 μm, 150 mm × 2.1 mm) column using an optimized acetonitrile: water gradient (both with 0.1% v/v of formic acid). The chromatographic, MS, and MS/MS conditions, as well as data processing, were conducted as described in previous works^12^. Detected compounds were annotated as metabolites considering their retention time (R_t_), accurate molecular mass, predicted molecular formula, and MS/MS spectra, through comparison with specific literature and various free databases, such as the Human Metabolome Database (http://www.hmdb.ca/*)*, PubChem (https://www.pubchem.ncbi.nlm.nih.gov/), ChemSpider (https://www.chemspider.com/, and the Phytochemical Dictionary of Natural Product Database (https://dnp.chemnetbase.com/faces/chemical/ChemicalSearch.xhtml*).* Consequently, the annotated metabolites were identified at a high confidence level (probable structure, level 2: MS, MS/MS, and bibliography/database^13^.

### In vitro AChE enzyme activity

The AChE enzyme activity of the methanol extract was assessed colorimetrically using an AChE enzyme inhibitor screening kit (BioVision, K197-100, Waltham, USA). Following a previously described method^14^, the AChE enzyme and the colorimetric substrate (5,5’-dithiobis (2-nitrobenzoic acid) solutions provided with the kit were mixed with 10 µL of the extract at different concentrations (0.1, 1.0, and 10.0 µg/mL in Tris-HCl buffer (pH 8.0)) in a 96-well microplate. The mixture was then incubated for 10–15 min at room temperature without exposure to light. Additionally, an enzyme-free blank and a DON (10 mM) positive standard were prepared. Absorbance was measured in all cases at 412 nm. Each analysis was carried out in triplicate, and the results were recorded as IC_50_ (the concentration inhibiting 50% of the target enzyme). Data were expressed as mean ± SD (*n* = 3).

### In vivo anti-AD activity

#### Animals

All male adult Wistar albino rats (200–250 g) were supplied by the National Research Centre (Giza, Egypt). They were housed in polypropylene cages under controlled environmental conditions of 55% ± 5% humidity and 25 ± 2 °C temperature. Before starting the study, the animals were given ad libitum access to a commercially available rat regular pellet diet and water for 7 days. The experiments adhered to the “Guide for the Care and Use of Laboratory Animals” published by the US National Institutes of Health (NIH Publication No 85–23, 2011). Approval was granted by the Research Ethics Committee of the Faculty of Pharmacy of Cairo University (approval code: MP (3118)).

#### Acute toxicity study

The lethal dose (LD_50_) of *C. infortunatum* extract (the concentration causing death to 50% of the tested group of animals) was determined in accordance with the guidelines outlined by the Organization for Economic Co-operation for Development (OECD, Test No 420, 2002). Thirty-six Rats, which had fasted overnight, were divided into six groups of six individuals. The extract, suspended in saline, was orally administered to the rats at doses of 125, 250, 500, 1000, and 2000 mg/kg body weight. The normal group was maintained under the same conditions and received a vehicle (saline). The animals were monitored over 48 h for signs of toxicity, and occurrences of death were recorded as mortality rates. Throughout the investigation, the rats had ad libitum access to food and water.

#### SCOP-induced AD study

The SCOP approach used in this study to induce AD was adapted from previous research^10^. Forty-eight male Wistar albino rats were divided into six groups of eight individuals, and the study extended over nine consecutive days. The healthy normal group (Group 1) received only saline. AD was induced in the remaining groups by intraperitoneal injection (IP) administration of SCOP at 5 mg/kg/day (daily for 9 days). Group 2 served as the SCOP-induced AD control group. The groups receiving the prophylactic treatments, after 30 min of SCOP, were orally administered the standard drug DON (2.5 mg/kg/day, daily for 9 days) or different doses of the methanol extract (100, 200, and 400 mg/kg/day, daily for 9 days). SCOP, DON, and the extracts were dissolved in saline using sonication to ensure complete dissolution. On day 8, the groups of rats were habituated and familiarized to the novel object (phases 1 and 2 of the NOR test). Memory was assessed on day 9 (phase 3) and rats were euthanized using thiopental (50 mg/kg; IP). The hippocampi of the eight rats from the group were isolated and homogenized in 10% phosphate-buffered saline for the enzyme-linked immunosorbent assay (ELISA) measurement of different biomarkers. The following biomarkers were measured: the memory function parameters (AChE enzyme activity and neurotransmitters ACh, DA, and NA), the oxidative stress parameters (GSH and MDA), the anti-inflammatory parameters (TNF-α and IL-1β), and the AD marker, Aβ protein. Rat ELISA kits from different manufacturers were used for the determination of the biomarkers, including assay kits from Abcam (Cambridge, UK) for AChE enzyme activity and Ach; from MyBioSource (San Diego, USA) for DA, NA, TNF-α, IL-1β, and Aβ; and from Lifespan Biosciences (Shirley, USA) for GSH and MDA. Finally, the brains were histopathologically evaluated. They were dissected out and kept in 10% neutral buffered formalin. After fixation, tissues were processed in alcohol and xylene. They were embedded in paraffin 5 μm thick, cut, and stained with hematoxylin and eosin^15^.

In the NOR test, a black open rectangular wooden box (45 × 65 × 45 cm) was used. The test was performed in a room with low noise levels and constant illumination. Two different opaque cubes served as familiar objects, and a pink pyramid was introduced as the novel object. These objects were 6 cm high and sufficiently heavy to prevent the rats from moving them. In addition, the objects were placed in opposite corners, positioned 10 cm away from the walls.

The NOR test involved three phases: Phase 1 (habituation phase): rats were individually allowed to explore the empty field for 5 min. Phase 2 (familiarization phase): rats were released into the open field and allowed to explore for 3 min two identical familiar objects (a_1_ and a_2_). Phase 3 (test phase): this phase was performed with the novel object (b) and the familiar object (a). The objects and open field were cleaned with 70% ethanol after each trial to minimize the presence of olfactory stimuli. Exploration was defined as rats directing their noses 2 cm from the object or touching it during the 3-minute test. After recording with a stopwatch, the time spent exploring the new object (t_b_) and the familiar object (t_a_), the discrimination index (DI) was determined (t_b_ - t_a_ / t_b_ + t_a_) %.

#### Statistical analysis

GraphPad Prism software (version 8; GraphPad Software, Inc., San Diego, USA) was used for graphical presentations and statistical analyses. All data were presented as mean ± standard deviation (SD). One-way ANOVA, followed by Tukey’s multiple comparison test, was conducted in the SCOP-induced AD study. A probability level of < 0.05 was considered statistically significant for all tests conducted.

## Results and discussion

### LC-QTOF-MS/MS metabolite profiling

**Fig. 1:**
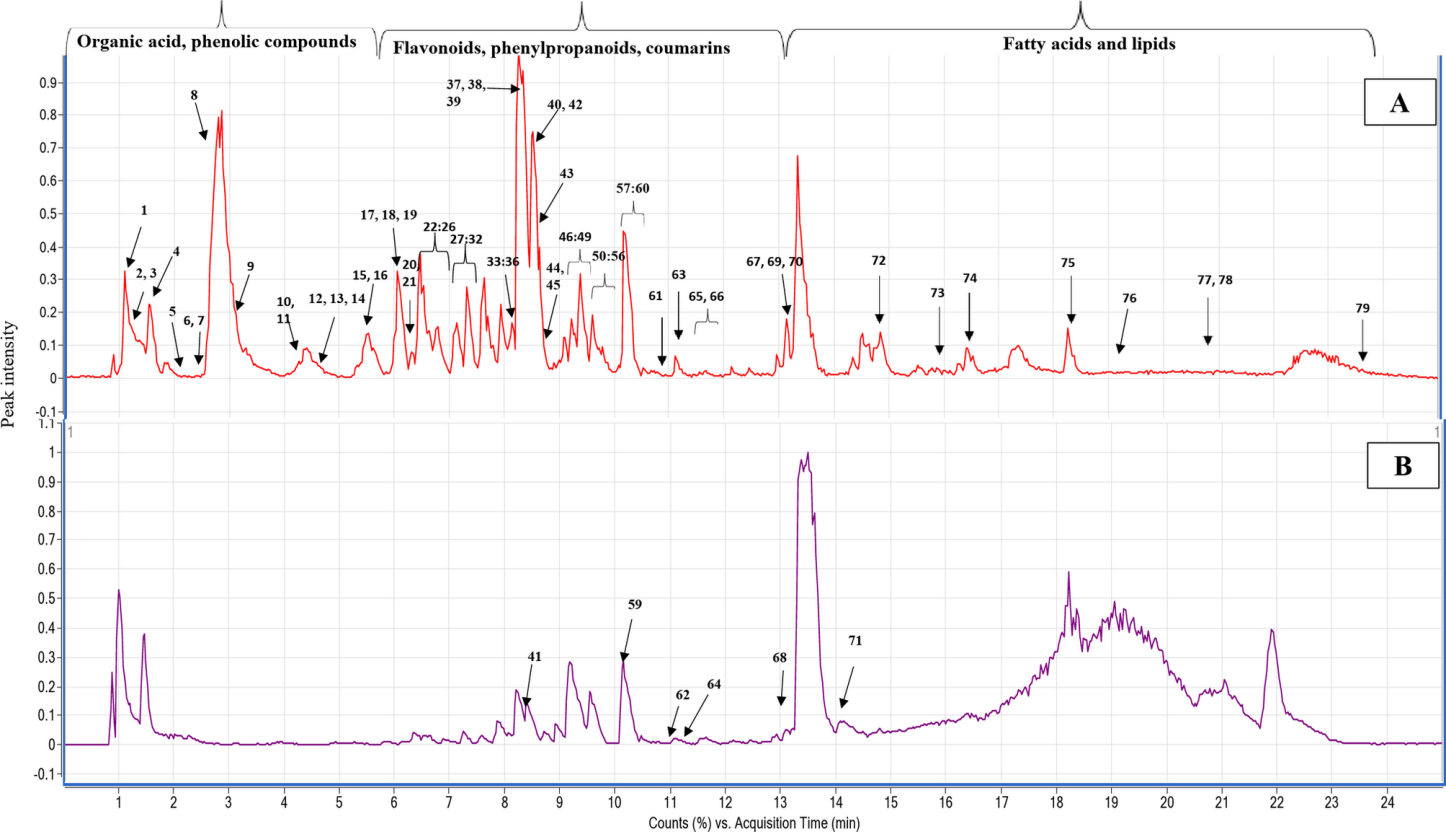
Representative LC-QTOF-MS/MS base peak chromatograms in (**A**) Negative ESI mode and (**B**) Positive ESI mode of the methanol extract of *C. infortunatum* aerial parts. The peak numbers of the identified metabolites are listed in Table 1.

The chemical profile of the methanol extract of *C. infortunatum* aerial parts was analyzed using LC-QTOF-MS/MS in the negative and positive ESI modes. The analysis enabled the comprehensive elution based on the polarity of plant constituents within 24 min, obtaining the first comprehensive metabolite profile of the methanol extract of *C. infortunatum* aerial parts. The representative base peak chromatograms of the methanol extract are shown in Fig. 1, and the MS/MS spectra of some of the most relevant metabolites are displayed in Fig. S1-S25. A total of 79 bioactive metabolites, detailed in Table 1 and belonging to various metabolite classes, were confidently annotated. These include 9 organic acids, 17 phenolic acids and other phenolics, 19 phenylpropanoids and phenylethanoids, 15 flavonoids, 4 coumarins, and 15 fatty acids and their derivatives. The interpretation of the mass spectra of some major metabolites is discussed below.

**Table 1:** Identified metabolites in the methanol extract of the *C. Infortunatum* aerial parts by LC-QTOF-MS/MS in the negative and positive ESI modes.

Peak no.	*R*_t_ (min)	Ion mode	m/z	Molecular formula	Error (ppm)	MS/MS	Identification	Reference
Organic acids								
1	1.16	[M-H] ^–^	133.0143	C_4_H_5_O_5_^–^	-0.4	115, 89, **71**, 73	Malic acid	^12^
2	1.4	[M-H] ^–^	115.0037	C_4_H_3_O_4_^–^	0.7	**71**	Fumaric acid	^16^
3	1.56	[M-H] ^–^	111.0088	C_5_H_3_O_3_^–^	-0.2	**78**, 67	Furoic acid	PubChem CID 10,268
4	1.57	[M-H] ^–^	191.0198	C_6_H_7_O_7_^–^	0.1	129, 111, **87**, 85	Citric acid	^17^
5	2.10	[M-H] ^–^	117.0193	C_4_H_5_O_4_^–^	2.8	**73**	Succinic acid	^18^
8	2.77	[M-H] ^–^	191.0563	C_7_H_11_O_6_^–^	3.1	129, 115, 111, **101**	Quinic acid	^18^
10	4.33	[M-H] ^–^	279.1086	C_11_H_19_O_8_^–^	-0.2	117, 99, 89, **59**	2-Hydroxy-2-methyl butyric acid hexoside	^19^
21	6.43	[M-H] ^–^	175.0614	C_7_H_11_O_5_^–^	-1.1	131, **115**, 85	Isopropyl malic acid	^12^
24	6.83	[M-H] ^–^	225.0407	C_10_H_9_O_6_^–^	-1.0	207, 163, **119**, 107	Chorismic acid	HMDB0012199
Phenolic acids								
9	3.12	[M-H]^–^	169.0140	C_7_H_5_O_5_^–^	1.4	147, **125**, 124, 115	Gallic acid	HMDB0005807
12	4.79	[M-H]^–^	197.0449	C_9_H_9_O_5_^–^	1.2	**135**, 123, 109, 72	Danshensu	HMDB0003503
13	4.82	[M-H]^–^	315.0724	C_13_H_15_O_9_^–^	-0.7	153, 152, 109, **108**	Protocatechuic acid hexoside	HMDB0303826
14	4.99	[M-H]^–^	167.035	C_8_H_7_O_4_^–^	3.4	**123**, 77, 55	Vanillic acid	^18^
15	5.48	[M-H]^–^	153.0195	C_7_H_5_O_4_^–^	-1.0	**109**, 91	Protocatechuic acid	HMDB0001856
17	6.11	[M-H]^–^	395.0986	C_15_H_19_O_10_^–^	-0.6	**197**, 153, 138, 96	Glucosyringic acid	HMDB0303364
20	6.361	[M-H]^–^	285.0617	C_12_H_13_O_8_^–^	-0.3	**152**, 109, 108	Catechol hexuronide	HMDB0240490
25	6.9	[M-H]^–^	325.0932	C_15_H_17_O_8_^–^	-0.9	163, **119**	Coumaric acid hexoside	^20^
26	6.92	[M-H]^–^	163.0397	C_9_H_7_O_3_^–^	-1.4	**119**, 101	*p*-Coumaric acid	^20^
27	7.21	[M-H]^–^	457.1351	C_20_H_25_O_12_^–^	-0.1	**163**, 119	Coumaric acid pentosyl hexoside	^19^
29	7.32	[M-H]^–^	473.1301	C_20_H_25_O_13_^–^	-0.1	**179**, 161, 135	Caffeic acid pentosyl hexoside	^19^
31	7.45	[M-H]^–^	179.035	C_9_H_7_O_4_^–^	-1.7	**135**, 117	Caffeic acid	^20^
52	9.64	[M-H]^–^	193.0507	C_10_H_9_O_4_^–^	-0.3	**161**, 134	Ferulic acid	^12^
Phenylpropanoids and phenylethanoids								
18	6. 21	[M-H]^–^	461.1664	C_20_H_29_O_12_^–^	0.1	315, 135, **113**	Decaffeoyl-acteoside )phenylethanoid glycoside(	^18^
19	6.25	[M-H]^–^	593.2079	C_25_H_36_O_16_^–^	1.3	461, **315**, 135	Pentosyl-decaffeoyl acteoside (phenylethanoid glycoside)	
22	6.5	[M-H]^–^	487.1459	C_21_H_27_O_13_^–^	-0.3	**179**, 161, 135	Cistanoside F (caffeoyl glycoside)	^18^
23	6.83	[M-H]^–^	341.0879	C_15_H_17_O_9_^–^	-0.2	221, **179**, 161, 135	1-*O*-Caffeoyl glucose	^21^
28	7.22	[M-H]^–^	501.1608	C_22_H_29_O_13_^–^	1.1	**193**, 175, 161, 134	Clemomandshuricoside B (feruloyl glycoside)	^22^
32	7.74	[M-H]^–^	639.1934	C_29_H_35_O_16_^–^	-0.5	621, 459, 179, **161**, 135	β-Hydroxy-verbascoside )caffeoyl phenylethanoid glycoside(	^18^
33	8.10	[M-H]^–^	785.2493	C_35_H_45_O_20_^–^	-0.8	623, 461, 315, **161**	Echinacoside (caffeoyl phenylethanoid glycoside)	^23^
35	8.15	[M-H]^–^	653.2089	C_30_H_37_O_16_^–^	-0.0	179, **161**, 135	Campneoside I (caffeoyl phenylethanoid glycoside)	^18^
36	8.21	[M-H]^–^	755.2412	C_34_H_43_O_19_^–^	-0.0	593, 461, **161**, 135, 89	Forsythoside B (caffeoyl phenylethanoid glycoside)	^18^
37	8.30	[M-H]^–^	623.1990	C_29_H_35_O_15_^–^	-1.3	461, 315, 179, **161**, 135, 113	Verbascoside (caffeoyl phenylethanoid glycoside)	^19^
40	8.56	[M-H]^–^	623.1990	C_29_H_35_O_15_^–^	-1.3	461, 315, 179, **161**	Iso-verbascoside )caffeoyl phenylethanoid glycoside(	^19^
42	8.68	[M-H]^–^	607.2034	C_29_H_35_O_14_^–^	-0.2	461, 315, 163, **161**	Lipedoside A-I )coumaroyl phenylethanoid glycoside(	^24^
43	8.87	[M-H]^–^	785.2291	C_39_H_41_O_18_^–^	0.9	623, **461**, 161, 89	Caffeoyl-verbascoside )caffeoyl phenylethanoid glycoside	
44	8.91	[M-H]^–^	637.2139	C_30_H_37_O_15_^–^	0.1	461, 315, 193, **175**	Leucosceptoside A )feruloyl phenylethanoid glycoside(	^25,26^
48	9.26	[M-H]^–^	783.2711	C_36_H_47_O_19_^–^	0.7	435, 355, 193, **175**, 160, 134	Angoroside C )feruloyl phenylethanoid glycoside(	^25^
49	9.38	[M-H]^–^	651.2295	C_31_H_39_O_15_^–^	-0.1	329, 193, **175**, 160, 113	Martynoside (feruloyl phenylethanoid glycoside (	^20^
50	9.45	[M-H]^–^	577.1923	C_28_H_33_O_13_^–^	0.6	179, **161**, 113	Salsaside A (caffeoyl phenylmethanoid glycoside)	^27^
56	9.98	[M-H]^–^	591.2085	C_29_H_35_O_13_^–^	-0.3	179, **161**	Jionoside C (caffeoyl phenylethanoid glycoside)	^25^
57	10.06	[M-H]^–^	693.2399	C_33_H_41_O_16_^–^	0.1	193, **175**, 160, 134	Acetyl martynoside (feruloyl phenylethanoid glycoside)	^25^
Flavonoids								
34	8.12	[M-H]^–^	637.1781	C_29_H_33_O_16_^–^	-1.0	**475**, 329, 193, 161, 110	Rhamnazin hexoside rhamnoside	^19^
38	8.45	[M-H]^–^	923.1526	C_42_H_35_O_24_^–^	-0.2	**285**, 113	Scutellarin derivatives	^28^
39	8.45	[M-H]^–^	461.0726	C_21_H_17_O_12_^–^	-0.1	**285**, 113, 57	Scutellarin	^20^
41	8.53	[M + H]^+^	287.0552	C_15_H_11_O_6_^+^	-0.6	**287**, 269, 169	Scutellarein	^18^
45	8.95	[M-H]^–^	431.0988	C_21_H_19_O_10_^–^	-0.9	311, 269, **268**	Apigenin hexoside	HMDB0041591
46	9.09	[M-H]^–^	445.0778	C_21_H_17_O_11_^–^	-0.3	**269**	Apigenin 7-glucuronide	^25^
47	9.20	[M-H]^–^	609.1261	C_30_H_25_O_14_^–^	2.9	**285**, 161, 135, 119	Kaempferol caffeoyl-hexoside	HMDB0030239
53	9.65	[M-H]^–^	475.0884	C_22_H_19_O_12_^–^	-0.4	**299**, 284, 113, 57	Hispidulin 7-glucuronide	^16^
54	9.67	[M-H]^–^	593.1293	C_30_H_25_O_13_^–^	1.2	**285**	Kaempferol coumaroyl-hexoside	^29^
55	9.93	[M-H]^–^	593.1293	C_30_H_25_O_13_^–^	1.2	269, **161**, 113	Apigenin caffeoyl-hexoside	PubChem CID 44,257,827
58	10.30	[M-H]^–^	459.0936	C_22_H_19_O_11_^–^	-0.6	**283**, 268, 85, 59	Acacetin 7-glucuronide	^19,25^
60	10.43	[M-H]^–^	577.1353	C_30_H_25_O_12_^–^	-0.2	**269**	Apigenin coumaroyl-hexoside	PubChem CID 44,257,855
61	10.87	[M-H]^–^	299.0552	C_16_H_11_O_6_^–^	0.3	**284**, 255, 227	4‵-Methyl scutellarein	^19^
63	11.15	[M-H]^–^	269.0458	C_15_H_9_O_5_^–^	-0.3	**269**, 225	Apigenin	^19^
67	13.00	[M-H]^–^	283.0615	C_16_H_11_O_5_^–^	-1.0	**268**	Acacetin	^16^
Coumarins								
30	7.43	[M-H]^–^	177.0194	C_9_H_5_O_4_^–^	-0.3	**133**, 105, 81	Esculetin	HMDB0030819
51	9.50	[M + H]^+^	485.1645	C_22_H_29_O_12_^+^	1.7	**177**, 145, 71	4-Methyl-umbelliferyl rhamnosyl-hexoside	
59	10.29	[M + H]^+^	339.1078	C_16_H_19_O_8_^+^	-1.0	277, 251, 177, **145**, 93	4-Methyl-umbelliferyl hexoside	
62	11.14	[M + H]^+^	177.0548	C_10_H_9_O_3_^+^	-1.0	162, 145,133, **117**	4-Methyl-umbelliferone	HMDB0059622
Other phenolics								
6	2.42	[M-H] ^–^	331.0707	C_13_H_15_O_10_^–^	-6.4	**331**, 169, 113, 96	Galloyl hexose	HMDB0301708
7	2.58	[M-H]^–^	125.0249	C_6_H_5_O_3_^–^	-3.8	**125**, 79	Pyrogallol or phloroglucinol	HMDB0013674 HMDB0013675
11	4.42	[M-H]^–^	153.0558	C_8_H_9_O_3_^–^	-0.5	**123**, 109	Syringol	HMDB0034158
16	5.54	[M-H]^–^	109.0295	C_6_H_5_O_2_^–^	0.0	**109**, 91	Catechol	^18^
Fatty acids and their derivatives								
64	11.5	[M + H]^+^	246.2430	C_14_H_32_NO_2_^+^	-0.0	**246**, 228	Tetradecasphinganine	PubChem CID 12,590,335
65	11.60	[M-H]^–^	327.2177	C_18_H_31_O_5_^–^	-0.0	291, 239, **185**	Trihydroxy-octadecadienoic acid	^12^
66	11.87	[M-H]^–^	329.2328	C_18_H_33_O_5_^–^	0.1	**249**, 229	Trihydroxy-octadecenoic acid	HMDB0038555
68	13.02	[M + H]^+^	333.2063	C_20_H_29_O_4_^+^	-0.8	**333**, 315, 107	Clerodermic acid	^19^
69	13.17	[M-H]^–^	311.2242	C_18_H_31_O_4_^–^	-4.4	**293**	Dihydroxy-octadecadienoic acid	^30^
70	13.18	[M-H]^–^	675.3604	C_33_H_55_O _14_^–^	-0.9	468, 415, 334, **277**, 59	Dihexosyl monoacyl glycerol (18:3)) (Ginger glycolipid A)	HMDB0041093
71	14.2	[M + H]^+^	272.2589	C_16_H_34_NO_2_^+^	-0.0	**254**, 100	Hexadecasphingosine	PubChem CID 14,767,871
72	14.81	[M-H]^–^	275.2175	C_18_H_27_O_2_^–^	0.5	**254**, 231, 79, 70	Octadecatetraenoic acid	HMDB0032672
73	15.95	[M-H]^–^	295.2277	C_18_H_31_O_3_^–^	0.5	**277**, 195, 171	Hydroxy-octadecadienoic acid	^19^
74	16.47	[M-H]^–^	293.2123	C_18_H_29_O_3_^–^	-0.2	**293**, 249, 191	Hydroxy-octadecatrienoic	^30^
75	18.32	[M-H]^–^	277.2175	C_18_H_29_O_2_^–^	-0.7	**277**, 233, 179	Octadecatrienoic acid (Linolenic acid)	HMDB0001388
76	19.29	[M-H]^–^	279.2328	C_18_H_31_O_2_^–^	0.5	**279**, 86, 59	Octadecadienoic acid (Linoleic acid)	^16^
77	20.70	[M-H]^–^	255.2323	C_16_H_31_O_2_^–^	-0.9	**255**, 211, 92	Hexadecanoic acid	^31^
78	20.9	[M-H]^–^	281.2485	C_18_H_33_O_2_^–^	0.3	**281**, 240	Octadecenoic acid (Oleic acid)	^16^
79	22.72	[M-H]^–^	819.5264	C_46_H_75_O_12_^–^	0.0	**353**, 277	Monohexosyl diacyl glycerol (18:3/18:3)	^12^

Numbers in bold represent the base peak.

#### Organic acids

It has been stated that organic acids, known for imparting sour or acidic taste to plants, significantly impact their organoleptic qualities^32^. Early elution time along with characteristic fragmentation patterns involving losses of H_2_O (-18) and CO_2_ (-44), aided in their annotation. For example, Peaks **4** [*m/z* 191.0198, C_6_H_7_O_7_^–^] and **8** [*m/z* 191.0563, C_7_H_11_O_6_^–^] were annotated as citric acid and quinic acid, respectively. Both peaks exhibited fragment ions at *m/z* 129, resulting from the successive losses of CO_2_ and H_2_O (Table 1 and Fig. S1 & S2), in accordance with the literature^18^.

#### Phenolic acids

Phenolic acids constitute a major group of secondary metabolites in plants, primarily categorized into two subgroups: hydroxybenzoic and hydroxycinnamic acids^20^. These compounds are typically identified by characteristic losses of CO_2_ (-44), CO (-28), H_2_O (-18), and CH_3_ (-14)^33^ (Table 1).

For instance, peaks **9** [*m/z* 169.0140, C_7_H_5_O_5_^–^], **15** [*m/z* 153.0195, C_7_H_5_O_4_^–^] and **26** [*m/z* 163.0397, C_9_H_7_O_3_^–^] displayed the loss of CO_2_ resulting in fragment ions at *m/z* 125, 109 and 119, respectively. They were identified as gallic acid, protocatechuic, and *p*-coumaric acid, respectively (Fig. S3-S5)^20^.

Peaks **27** [*m/z* 457.1351, C_20_H_25_O_12_^–^] and **29** [*m/z* 473.1301, C_20_H_25_O_13_^–^] were annotated as phenolic acid glycosides. Their assignment was confirmed by the loss of the attached pentose-hexose unit [M–162–132]^–^, yielding fragment ions at *m/z* 163 and 179, respectively. Accordingly, metabolites **27** and **29** were identified as coumaric acid pentosyl hexoside and caffeic acid pentosyl hexoside, respectively (Fig. S6 & S7)^19^.

#### Phenylpropanoids and phenylethanoids

Phenylpropanoids represent the major components of *C. infortunatum* (Table 1). They display a variety of pharmacological properties such as anti-inflammatory, antimicrobial, and anti-skin-aging effects^34^. The phenylpropanoid, primarily caffeic acid, can potentially bind with sugars forming ester in association with a phenylethanoid moiety. In detail, peaks **32** [*m/z* 693.1934, C_29_H_35_O_16_^–^], **36** [*m/z* 755.2412, C_34_H_43_O_19_^–^], and **37** [*m/z* 623.1990, C_29_H_35_O_15_^–^] revealed their base peak fragment at *m/z* 161 for caffeoyl moiety, in addition to other ions corresponding to the loss of the attached sugar residues. Eventually, metabolites **32**, **36**, and **37** were annotated as β-hydroxy-verbascoside, forsythoside B, and verbascoside (Fig. S8-S10)^18,19^. Following the same fragmentation pattern, metabolites **48** [*m/z* 783.2711, C_36_H_47_O_19_^–^], **49** [*m/z* 651.2295, C_31_H_39_O_15_^–^], and **57** [*m/z* 693.2399, C_33_H_41_O_16_^–^] presented their base peak fragment at *m/z* 175 for feruloyl moiety, along with additional fragment ions corresponding to the loss of the attached sugar residues. They were identified as angoroside C, martynoside, and acetyl martynoside (Fig. S11-S13).

#### Flavonoids

Flavonoids are secondary metabolites that perform many functions, like regulating cell growth, attracting pollinators and insects, and protecting against biotic and abiotic stresses^35^. Because of their bioactive characteristics, these substances have been linked to a wide range of health advantages in people, including antidiabetic, cardio-protective, neuroprotective, antiviral, antibacterial, and anti-aging effects^36^. The extract was found enriched in flavonols (mainly kaempferol and rhamnazin glycosides) and flavones (apigenin, hispidulin, and acacetin derivatives) (Table 1). Flavonoid glycosides commonly occur as *O*-glycosides where the sugar moieties are linked to the flavonoid aglycone (Ag) through an *O*-glycosidic bond. This can be readily identified in tandem mass spectrometry, according to the neutral loss(es) of the attached sugar, whereby − 162, -146, and − 132 mass units indicate the loss of hexose, deoxyhexose (rhamnose), and pentose moieties, respectively^37^. A flavonol glycosylated with two sugar moieties, peak **34** [*m/z* 637.1781, C_29_H_33_O_16_^–^], showed fragment ions 475 and 329 due to loss of hexose followed by rhamnose, respectively, and was annotated as rhamnazin hexoside-rhamnoside (Fig. S14)^19^. Flavonoids **39** [*m/z* 461.0726, C_21_H_17_O_12_^–^], **46** [*m/z* 445.0778, C_21_H_17_O_11_^–^], **53** [*m/z* 475.0884, C_22_H_19_O_12_^–^] and **58** [*m/z* 459.0936, C_22_H_19_O_11_^–^] revealed base peak fragments at *m/z* 285, 269, 299, and 283, respectively, due to loss of the attached sugar unit [M–176]^–^. They were consequently identified as scutellarin, apigenin 7-glucuronide, hispidulin 7-glucuronide, and acacetin 7-glucuronide, respectively (Fig. S15-S18)^16,25^. Among other flavonoids annotated, showing a conjugation between sugar and cinnamoyl moieties (Figs. S19-S22), there were kaempferol caffeoyl-hexoside, **47** [*m/z* 609.1261, C_30_H_25_O_14_^–^]; kaempferol coumaroyl-hexoside, **54** [*m/z* 593.1293, C_30_H_25_O_13_^–^]; apigenin caffeoyl-hexoside, **55** [*m/z* 593.1293, C_30_H_25_O_13_^–^]; and apigenin coumaroyl-hexoside, **60** [*m/z* 577.1353, C_30_H_25_O_12_^–^]^38^.

#### Coumarins

Coumarins are an important and widely distributed group of natural phytochemicals, representing a class of lactones structurally constructed by a benzene ring fused to an α-pyrone ring^39^. Three umbelliferone compounds were detected in the investigated samples, namely, 4-methyl-umbelliferyl rhamnosyl-hexoside (**51**) [*m/z* 485.1645, C_22_H_29_O_12_^+^], 4-methyl-umbelliferyl hexoside (**59**) [*m/z* 339.1078, C_16_H_19_O_8_^+^], and 4-methyl-umbelliferone (**62**) [*m/z* 177.0548, C_10_H_9_O_3_^+^]. Peaks **51** and **59** showed an intense ion at *m*/*z* 177 corresponding to the 4-methyl-umbelliferone moiety after the detachment of linked sugar(s) (Fig. S23 & S24 & S25).

#### Fatty acids and their derivatives

Fatty acids represent the primary major structural elements of lipids and the fundamental building block of cell membranes^40^. A total of 15 fatty acids or their derivatives, were observed in the chromatogram (Fig. 1; Table 1). The primary process of fatty acid fragmentation involved neutral water loss(es) and decarboxylation^32^. Examples of identified hydroxylated fatty acids were peaks **65**, **66**, **73**, and **74**. Peak **65** [*m/z* 327.2177, C_18_H_31_O_5_^–^] can be distinguished from peak **66** [*m/z* 329.2328, C_18_H_33_O_5_^–^], by a difference of + 2 mass units, indicating the existence of an extra double bond in the former. These two peaks were tentatively identified as trihydroxy-octadecadienoic acid and trihydroxy-octadecenoic acid. Similarly, peaks **73** [*m/z* 295.2277, C_18_H_31_O_3_^–^] and **74** [*m/z* 293.2123, C_18_H_29_O_3_^–^] were identified as hydroxy-octadecadienoic acid and hydroxy-octadecatrienoic, respectively (Table 1). Additionally, fatty acid **75** [*m/z* 277.2175, C_18_H_29_O_2_^–^] exhibits a difference of -16 mass units compared to **74** suggesting the presence of an additional hydroxyl group in the latter, and was annotated as octadecatrienoic acid (linolenic acid) (Table 1).

Lipid synthesis occurs through the esterification of fatty acids on the glycerol skeleton. Most lipids are insoluble in water and fall into a number of groups, including sulfolipids, glycolipids, and sphingolipids^32^. In the negative ESI mode, the presence of carboxylate ions [RCOO] − enables the identification of individual fatty acids esterified on the glycerol skeleton. For example, glycolipids **70** [*m/z* 675.3604, C_33_H_55_O_14_^–^] and **79** [*m/z* 819.5264, C_46_H_75_O_12_^–^] both show a fragment ion at *m/z* 277 for octadecatrienoic moiety. Consequently, they were annotated as dihexosyl monoacyl glycerol (18:3) and monohexosyl diacyl glycerol (18:3/18:3), respectively (Table 1).

### In vitro AChE enzyme activity

The inhibitory effect on AChE enzyme activity was assessed colorimetrically for the extract, revealing a dose-dependent reduction in AChE enzyme activity. The extract and DON significantly inhibited the AChE enzyme activity compared to the control, with IC_50_ values of 0.18 ± 0.01 and 0.08 + 0.00 µg/mL, respectively.

### In vivo anti-AD activity

#### Acute toxicity study

The term “toxicity” refers to a substance’s ability to harm humans or animals, as well as a description of the effect and the concentration at which the effect occurs^41^. Toxicity is often estimated by LD_50_ at 24 h. In the present study, no mortality nor signs of toxicity were observed even at a dose as high as 2000 mg/kg of the extract over 48 h. Consequently, the selected dose for further investigation was set at 400 mg/kg.

#### **SCOP**-**induced AD study**

**Fig. 2:**
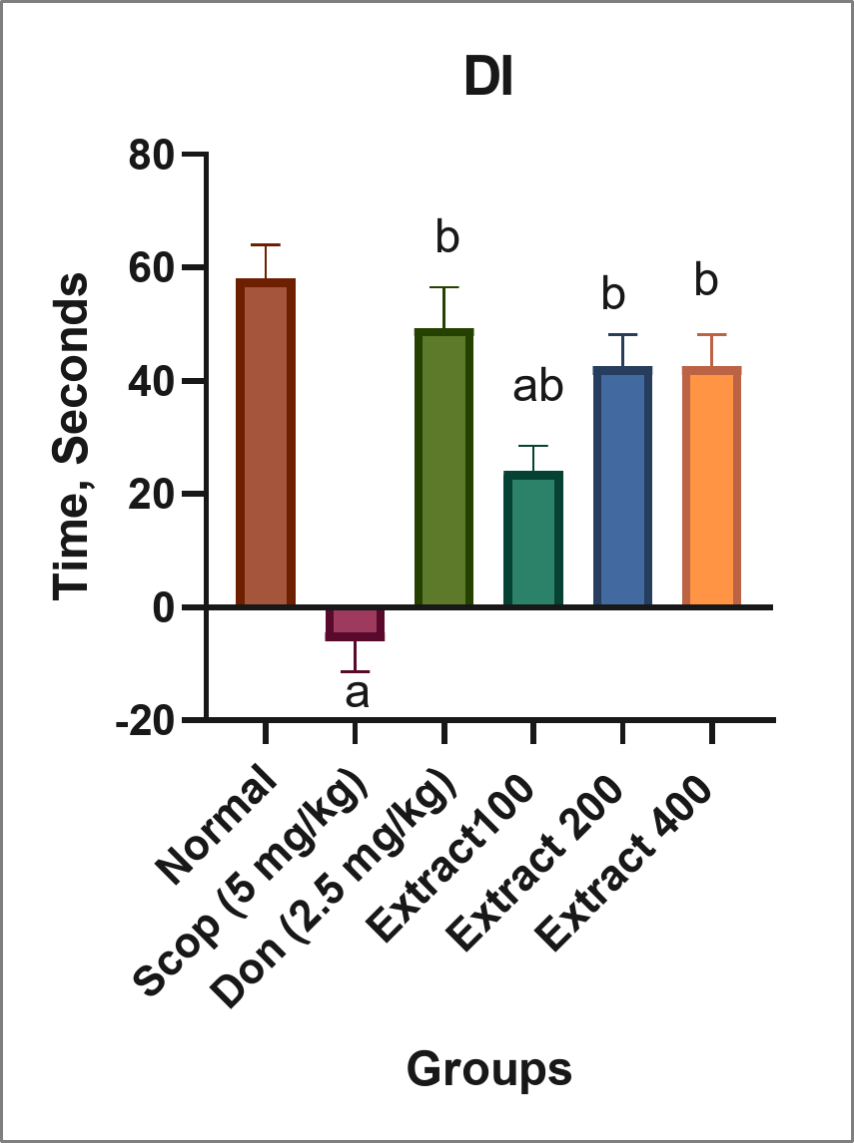
NOR test discrimination index (DI) values in the studied groups of rats. The normal group was not treated with SCOP. The rest of the groups were treated with SCOP to induce AD, and in some cases also with DON (2.5 mg/kg) or extract (100, 200, and 400 mg/kg). Values are expressed as mean ± SD (*n* = 8). Different inset letters indicate statistically significant differences (*p* < 0.05) compared to the: (a) normal group, (b) SCOP-induced AD group, and ab. normal and SCOP-induced AD groups.

The NOR test was performed to assess the cognitive function and memory of rats, relying on the natural inclination of rodents to explore novel objects. As shown in Fig. 2, SCOP-induced AD rats took less time to identify the novel object, compared to the normal group, indicating deteriorated neurocognitive function^42^. Additionally, DON and the extract significantly increased the time rats spent examining the novel object compared to the SCOP-induced AD group. Specifically, the DI for the groups receiving DON, as well as 400, 200, and 100 mg/kg extract were 49, 42, 42, and 24 s, respectively. The most notable impact was observed in the DON group followed by the 400 mg/kg and 200 mg/kg extract groups. All these groups displayed significant differences when compared to the SCOP-induced group and minimal non-significant differences in comparison to the normal group.

In SCOP-induced AD rats, the brain level of ACh was significantly decreased by 82% compared to the normal group (Table 2), consistent with previous findings^43^. Rats treated with DON, 200 mg/kg extract, and 400 mg/kg extract showed significantly higher levels of ACh, compared to the SCOP-induced AD group, with increases of 329%, 172%, and 287%, respectively. In contrast, the AChE enzyme activity and levels of DA and NA in SCOP-induced AD rats were elevated by 352%, 317%, and 375%, respectively, compared to the normal group, aligning with reported data^43^. On the other hand, both DON and the extract at certain concentrations lowered brain AChE enzyme activity, as well as DA and NA levels. The most significant improvement, compared to the SCOP-induced AD group, was observed in the DON group, followed by the 400 and 200 mg/kg extract groups. Specifically, DON significantly reduced AChE enzyme activity, as well as DA and NA levels, with reductions of 81%, 73%, and 71%, respectively. Similarly, the 400 mg/kg extract group displayed significant reductions of 76%, 64%, and 66%, respectively, while the 200 mg/kg group showed slightly lower values at 55%, 46%, and 47%, respectively.

**Table 2:** Evaluation of the memory function parameters (AChE enzyme activity, ACh, DA, and NA levels) in the studied groups of rats. The normal group was not treated with SCOP. The rest of the groups were treated with SCOP to induce AD, and in some cases also with DON (5 mg/kg) or methanol extract (100, 200, and 400 mg/kg).

Parameter	Normal	SCOP (5 mg/kg)	DON (2.5 mg/kg)	Extract (100 mg/kg)	Extract (200 mg/kg)	Extract (400 mg/kg)
ACh (nmol/mg)	5.17 ± 0.5	0.93^a^ ± 0.2	3.98^b^ ± 0.3	1.28^a^ ± 0.2	2.52^ab^ ± 0.2	3.52^b^ ± 0.2
AChE enzyme activity (U/mg.tissue)	0.20 ± 0.0	0.90^a^ ± 0.0	0.17^b^ ± 0.02	0.81^a^ ± 0.1	0.40^ab^ ± 0.0	0.22^b^ ± 0.0
DA (ng/mg.tissue)	12.10 ± 1.4	50.50^a^ ± 2.3	13.41^b^ ± 2.3	36.63^ab^ ± 4.7	27.17^ab^ ± 4.3	18.17^ab^ ± 2.3
NA (pg/mg.tissue)	5.70 ± 0.9	27.07^a^ ± 4.3	7.91^b^ ± 0.8	21.25^a^ ± 3.1	14.27^ab^ ± 1.9	9.32^b^ ± 1.7

Data are expressed as mean ± SD (*n* = 8). Different inset letters indicate statistically significant differences (*p* < 0.05) compared to the: ^a^Normal group, ^b^SCOP-induced AD group, and ^ab^Normal and SCOP-induced AD groups.

**Fig. 3:**
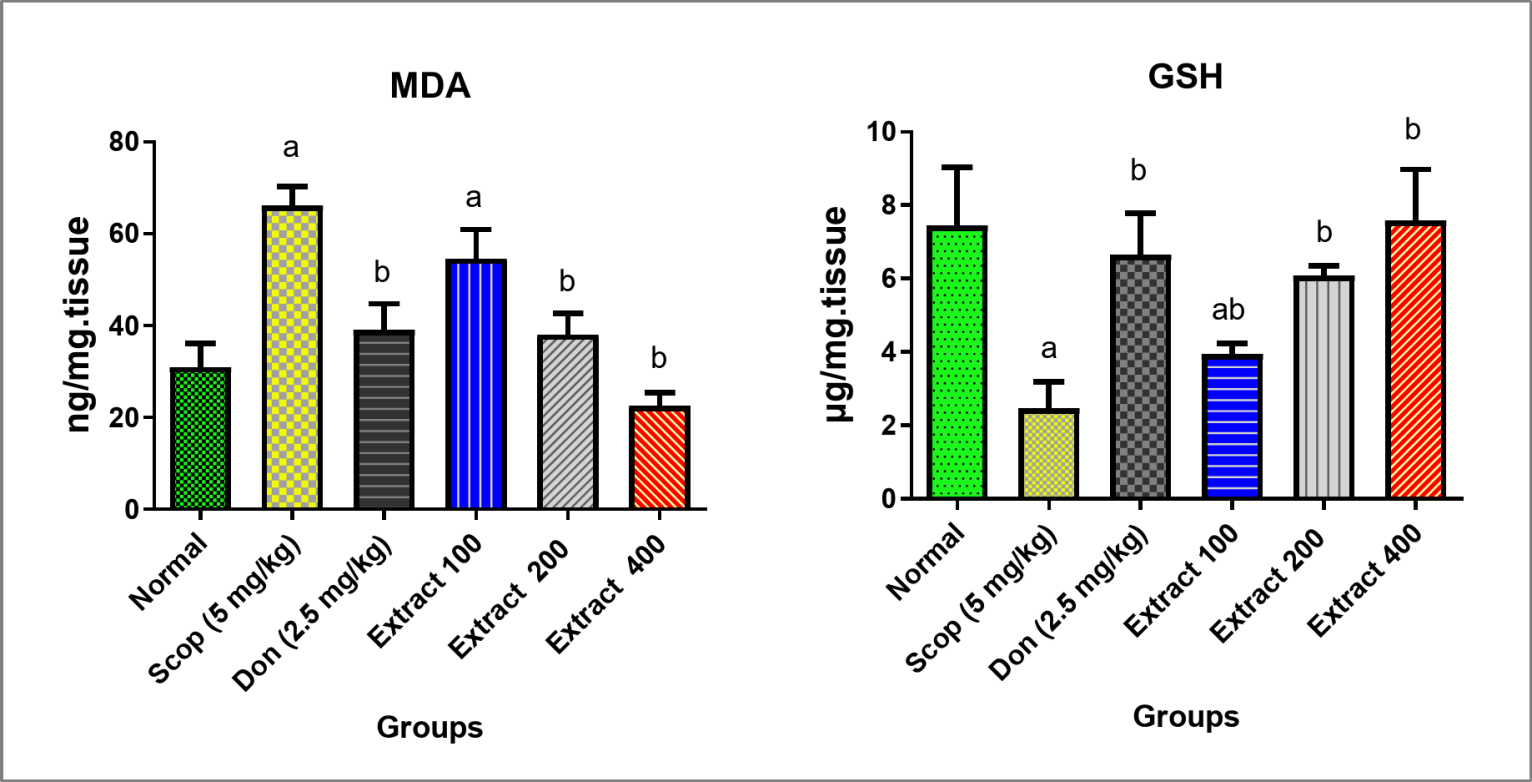
Evaluation of the oxidative stress parameters (MDA and GSH levels) in the studied groups of rats. The normal group was not treated with SCOP. The rest of the groups were treated with SCOP to induce AD, and in some cases also with DON (2.5 mg/kg) or total extract (100, 200, and 400 mg/kg). Parameters are expressed as mean ± SD (*n* = 8). Different inset letters indicate statistically significant differences (*p* < 0.05) compared to the: (a) normal group, (b) SCOP-induced AD group, and ab. normal and SCOP-induced AD groups.

In terms of oxidative stress parameters, SCOP administration is expected to result in the elevation of hippocampal MDA content, the final product of lipid peroxidation and one of the most frequently used markers for damage caused by free radicals^44^. This is accompanied by a subsequent reduction in the endogenous antioxidant GSH^44^. Accordingly, the MDA level was significantly higher in the SCOP-induced AD group, showing a 113% increase compared to the normal group (Fig. 3). Concurrently, GSH levels in the SCOP-induced AD group were significantly lower, showing a 67% decrease compared to the normal group, as described in previous works^43^. Administration of DON or various concentrations of extract to rats demonstrated potent antioxidant properties by lowering MDA levels and elevating GSH levels in brain tissues. Notably, the group treated with 400 mg/kg of extract exhibited the most significant improvements, with a reduction in MDA levels by 66% and an increase in GSH levels by 208% compared to the SCOP-induced AD rat group. Following closely were the DON and 200 mg/kg extract groups, which showed reductions in MDA levels by 41% and 42%, and increases in GSH levels by 170% and 147%, respectively. The 100 mg/kg group also showed a significant increase in GSH levels compared to the SCOP-induced AD group, demonstrated comparatively lesser effects. These results suggested that the anti-AD protective effect of *C. infortunatum* extract on SCOP-induced AD rats may be partly due to its antioxidant activity.

**Fig. 4:**
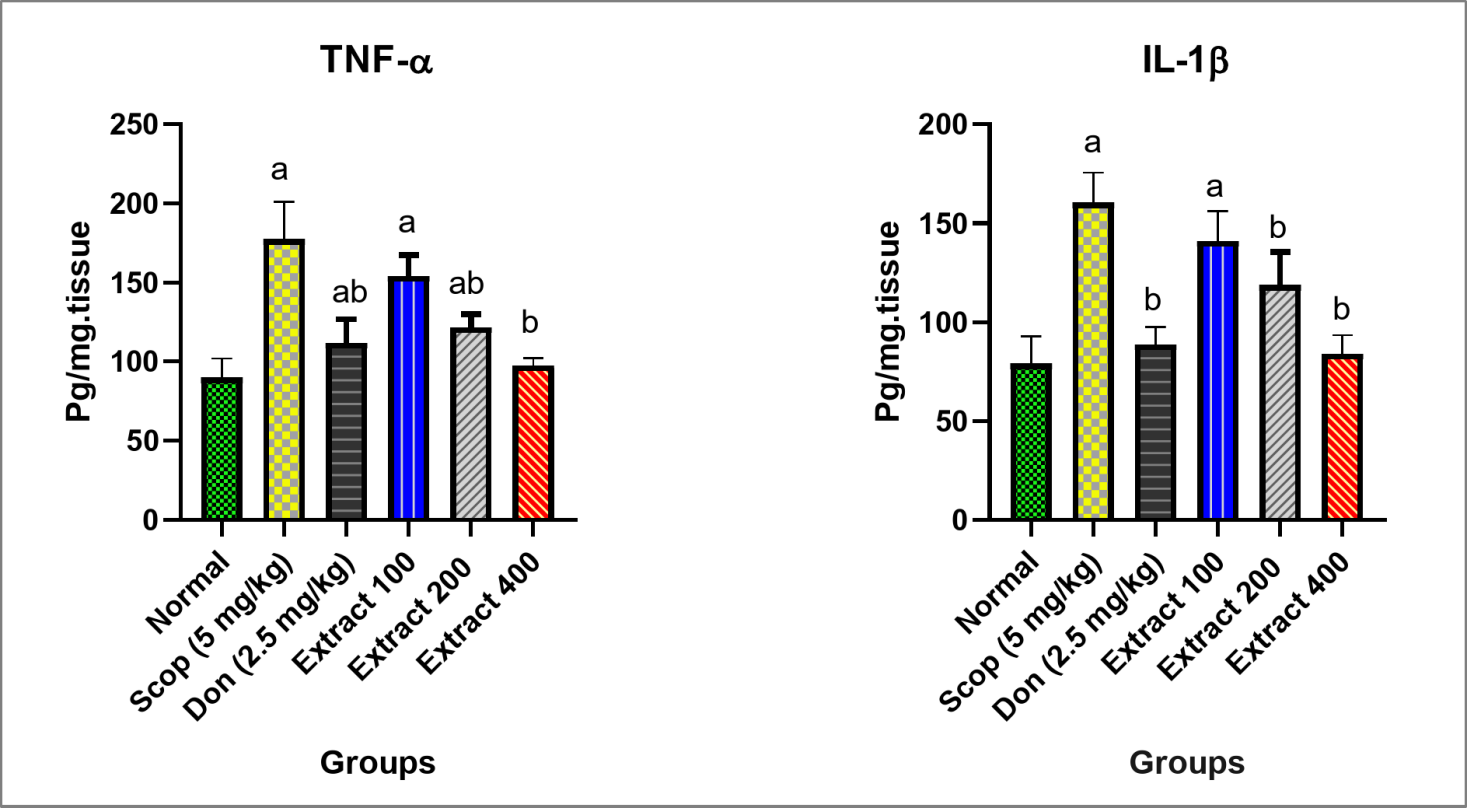
Evaluation of the anti-inflammatory parameters (TNF-α and IL-1β levels) in the studied groups of rats. The normal group was not treated with SCOP. The rest of the groups were treated with SCOP to induce AD, and in some cases also with DON (2.5 mg/kg) or total extract (100, 200, and 400 mg/kg). Parameters are expressed as mean ± SD (*n* = 8). Different inset letters indicate statistically significant differences (*p* < 0.05) compared to the: (a) normal group, (b) SCOP-induced AD group, and ab. normal and SCOP-induced AD groups.

Additionally, the increase in ROS resulting from oxidative stress is widely recognized for triggering the activation of pro-inflammatory cytokines, such as TNF-α and IL-1β^45^. In SCOP-induced AD rats, the levels of TNF-α and IL-1β were significantly higher by 97% and 103%, respectively, compared to the normal group (Fig. 4). These elevated levels persisted with the treatment at a dose of 100 mg/kg of extract. Notably, the 200 mg/kg and 400 mg/kg extract, and DON groups exhibited significantly decreased levels of TNF-α, with reductions of 32%, 45%, and 37%, respectively, compared to the SCOP-induced AD group. Similarly, IL-1β levels in these groups showed reductions of 26%, 48%, and 45%, respectively. This suggested that the extract ameliorated cognitive deficits by suppressing the inflammatory cascade, probably through its antioxidant effect. This finding regarding the anti-inflammatory activity of *C. infortunatum* was consistent with previously reported research^46^.

**Fig. 5:**
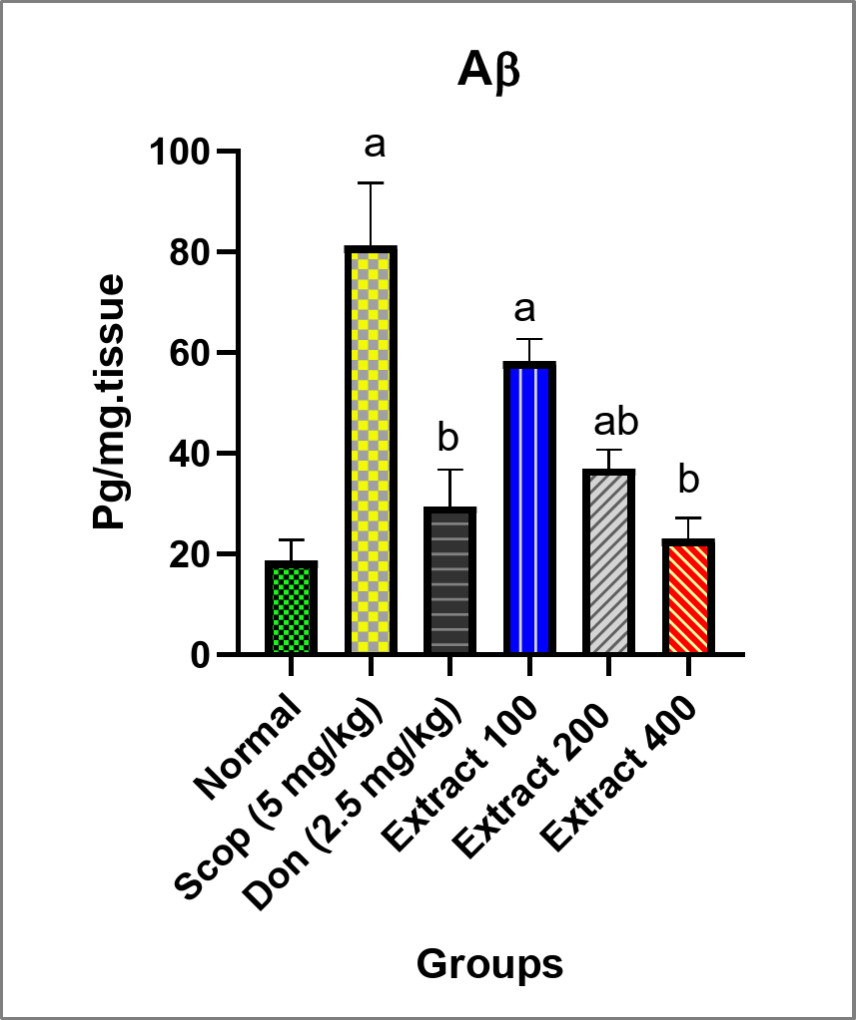
Evaluation of the anti-Aβ protein level in the studied groups of rats. The normal group was not treated with SCOP. The rest of the groups were treated with SCOP to induce AD, and in some cases also with DON (2.5 mg/kg) or total extract (100, 200, and 400 mg/kg). Parameters are expressed as mean ± SD (*n* = 8). Different inset letters indicate statistically significant differences (*p* < 0.05) compared to the: (a) normal group, (b) SCOP-induced AD group, and ab. normal and SCOP-induced AD groups.

Aβ protein, a β-sheet protein, is derived from the Aβ precursor protein through the activities of β and γ-secretase. The clinical manifestation of AD is associated with multiple accumulations of senile plaques composed of Aβ protein^47^. In SCOP-induced AD rats, a significant increase in Aβ protein levels by 337% was observed compared to the normal group, consistent with the reported findings^48^. Remarkably, Aβ protein levels in groups treated with 200 mg/kg extract, 400 mg/kg extract, and DON were significantly reduced by 54%, 72%, and 64%, respectively, compared to the SCOP-induced AD group (Fig. 5). Therefore, the extract showed an anti-AD protective effect by decreasing Aβ in the hippocampus.

**Fig. 6:**
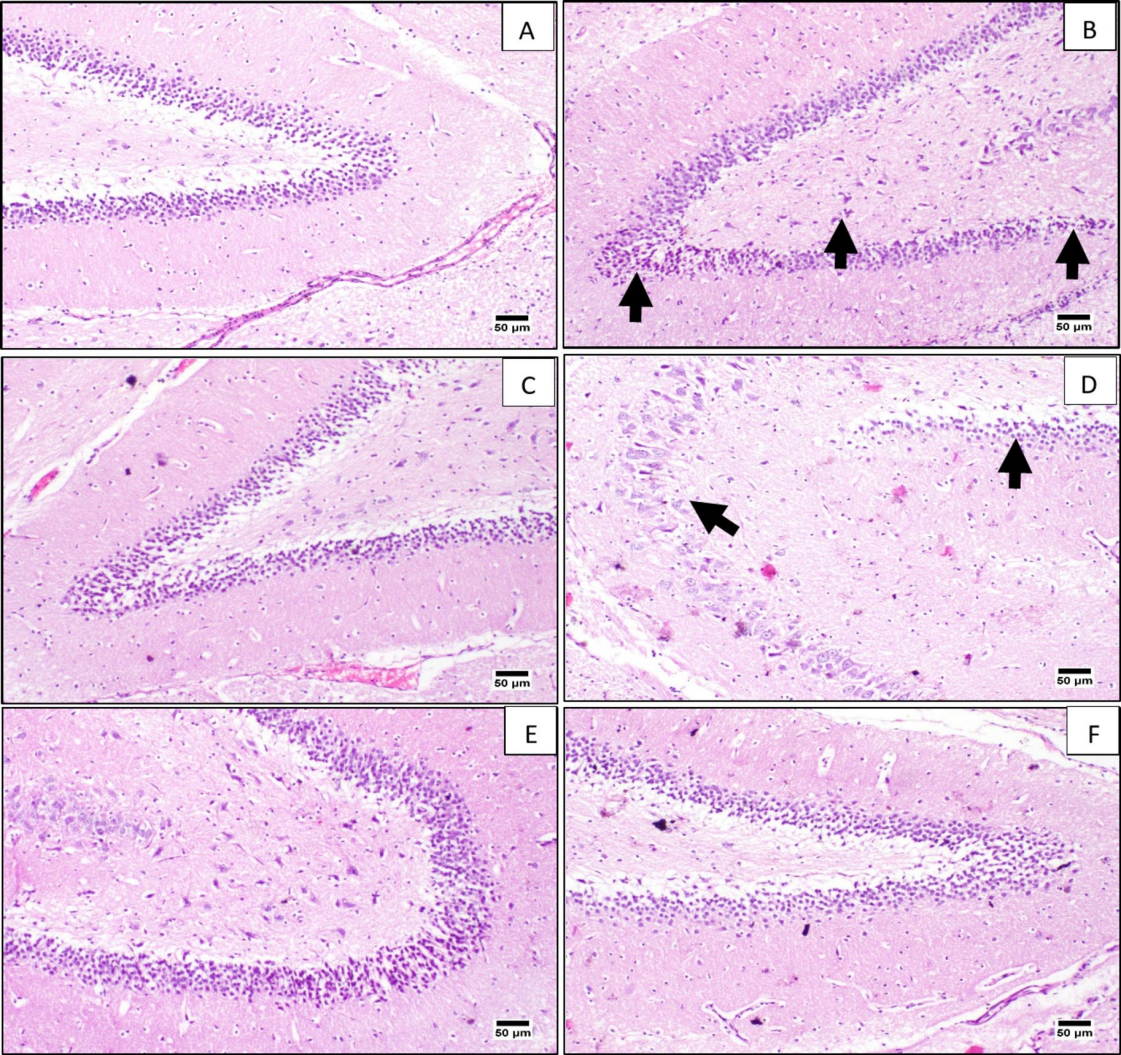
Histopathological evaluation of the studied groups of rats (*n* = 8). (**A**) Normal group was not treated with SCOP and showed the typical hippocampus region. The rest of the groups were treated with SCOP to induce AD. (**B**) SCOP group, with no preventive treatment, showed neuronal degeneration (arrows), (**C**) DON group showed almost normal neurons, (**D**) 100 mg/kg methanol extract showed diffuse mild neuronal gliosis, (**E**) 200 mg/kg methanol extract, and (**F**) 400 mg/kg methanol extract both showed apparently normal neurons.

As a complement to the molecular biomarker analyses, the results of the histopathological evaluation are displayed in Fig. 6. Microscopic examination of the hippocampi of the normal group (Fig. 6A), DON (Fig. 6C), 200 mg/kg (Fig. 6E), and 400 mg/kg extract (Fig. 6F) groups revealed the normal histological architecture of the hippocampus compared to the normal group. In contrast, hippocampal sections of SCOP-induced AD rats (Fig. 6B) and 100 mg/kg of extract (Fig. 6D) exhibited numerous histopathological changes due to neuronal degeneration (arrows in Fig. 6B), including diffuse mild gliosis (arrows in Fig. 6D).

Metabolite profiling of *C. infortunatum* extract revealed the presence of many phytochemicals, which may explain its observed anti-AD bioactivity. These phytochemicals include organic acids, phenolic acids, phenylpropanoids and phenylethanoids, flavonoids, coumarins, other phenolics, and fatty acids and their derivatives. Notably, flavonoids, phenylpropanoids, and fatty acids emerged as major metabolites, considering their peak intensities, all showing promising anti-AD properties^49–51^. To the best of our knowledge, this is the first report highlighting the involvement of brain neurotransmitters, cholinergic activity, oxidative stress biomarkers (MDA and GSH), inflammatory biomarkers such as TNF-α and IL-1β, as well as histopathological changes in the anti-AD effect of *C. infortunatum* in SCOP-induced AD rats. Additionally, cognitive function, including memory and recognition, was assessed using the NOR test.

In this study, SCOP-induced AD rats exhibited impaired recognition memory in the NOR test, as they explored both familiar and novel objects similarly and were unable to discriminate between the two. These results align with previous studies^43^. Interestingly, *C. infortunatum* -treated rats reversed SCOP-induced AD in the NOR test as they were able to discriminate between the familiar and novel objects and spent more time identifying the novel object relative to the familiar one. These effects were comparable to those observed with the standard drug, DON. Moreover, SCOP administration resulted in cholinergic system dysfunction as evidenced by elevated AChE activity and a reduction in ACh, an essential neurotransmitter involved in learning and memory. This finding is consistent with prior studies^43^. Preventive treatment with *C. infortunatum* and DON decreased hippocampal AChE activity and increased ACh levels, indicating that *C. infortunatum* might have ameliorated the cognitive deficits observed in the NOR test partly by enhancing cholinergic neurotransmission.

The role of organic and phenolic acids in memory enhancement has been well-documented. Quinic acid (**8**, Table 1), administered orally at doses of 200 mg/kg and 400 mg/kg, normalized AChE activity and mitigated the behavioral deficits in aluminum-induced AD rats^52^. Similarly, caffeic acid (**31**, Table 1), whether in its free form or combined with other groups like quinic acid and sugars, exhibits notable brain effects, including protection against AD-induced models^53^.

Additionally, neurotransmitters such as DA and NA play significant roles in memory retrieval^10^. SCOP administration increased DA and NA levels in AD-induced rats, consistent with previous research^10^, while *C. infortunatum* administration restored these neurotransmitters to normal levels, similar to DON. These findings underscore the potential of *C. infortunatum* in modulating neurotransmitter activity to alleviate AD.

Oxidative stress has been implicated in the pathogenesis of AD. This was evident in the current investigation, as SCOP-induced AD resulted in elevated MDA content and a subsequent reduction in the endogenous antioxidant GSH, likely due to increased ROS levels. This finding aligns with a prior study^43^ and suggests that oxidative stress associated with SCOP contributes to memory impairment. Interestingly, preventive treatment with *C. infortunatum* reversed SCOP-associated oxidative stress, bringing it to levels similar to DON by reducing MDA and enhancing the ROS-scavenging activity of GSH. Previous studies on *Clerodendrum speciosum* revealed its antioxidant effects^38^, which may also apply to *C. infortunatum*. This reveals that the ameliorative effects of natural compounds like flavonoids as apigenin (**63**, Table 1) on SCOP-induced AD may partly be due to their ability to restore oxidative balance by increasing GSH and decreasing MDA^54^.

Furthermore, SCOP increased the expression of inflammatory biomarkers such as TNF-α and IL-1β, suggesting that elevated inflammation may contribute to cognitive deficits in SCOP-induced AD rats^43,44^. Previous studies^46,55^ have demonstrated the anti-inflammatory effects of *C. infortunatum* in inflamed rat models. This suggests that *C. infortunatum* may ameliorate cognitive deficits through the suppression of inflammatory biomarkers, likely *via* its antioxidant properties.

Additionally, SCOP-induced AD resulted in a significant elevation of Aβ protein, a well-established biomarker of AD pathology. This finding aligns with previous studies^48^ and suggests that Aβ accumulation exacerbates neuronal damage and cognitive decline by forming neurotoxic plaques and initiating inflammatory responses. Administration of *C. infortunatum* significantly reduced Aβ protein levels, similar to DON, indicating its neuroprotective effects.

The neuroprotective effects of *C. infortunatum* extract can be attributed primarily, to its abundant content of phenylpropanoids and flavonoids, which exhibit potent antioxidant and anti-inflammatory activities. For instant, echinacoside (**33**, Table 1) has been found to exhibit protective activity in neurodegenerative disease by reducing ROS production, glia cell activation, Aβ deposition, and pro-inflammatory cytokine IL-1β, and TNF-α release^56^. Forsythoside B (**36**, Table 1) inhibits inflammatory mediators like TNF-α and IL-β, which are significant contributors to AD pathogenesis^57^. Moreover, verbascoside (**37**, Table 1) enhances memory and spatial cognition by suppressing Aβ deposition and tau protein accumulation^49^. Additionally, the protective effects of scutellarin (**41**, Table 1) in AD, both in vitro and in vivo, are attributed to its antioxidant and anti-inflammatory properties. Scutellarin has been shown to enhance the levels of ACh and reduce levels of ROS in the brain, leading to decreased Aβ deposition^58^. Similarly, Apigenin (**63**, Table 1) is known for its potent anti-inflammatory effects involving the downregulation of cytokines and nitric oxide, thereby preserving neurite integrity and cell viability^50^.

In addition, coumarins as intriguing acetylcholinesterase and butyrylcholinesterase inhibitors, have been shown to mitigate oxidative stress and neuroinflammation^59^. These dual actions align closely with the neuroprotective properties of fatty acids, particularly linolenic and oleic acids, which play a crucial role in mitigating AD progression. For instance, linolenic acid (**76**, Table 1) shows potential in regulating inflammatory responses within the central nervous system, the main site of inflammation in AD. Additionally, it has demonstrated efficacy as a dietary AChE inhibitor, thereby enhancing its neuroprotective properties^60^. On the other hand, oleic acid (**78**, Table 1), which is abundant in neuronal myelin sheaths, contributes to membrane phospholipid integrity and has been associated with reduced risk of cancer and AD, alongside cholesterol-lowering benefits^61^. Notably, lower levels of oleic acid have been observed in individuals with major depressive disorders and AD cases^51^.

Despite the significant potential of certain metabolites present in the *C. infortunatum* extract, further studies are needed to isolate and evaluate individual bioactive metabolites or enriched fractions. These findings underscore the multifaceted therapeutic potential of natural compounds from the aerial parts of *C. infortunatum* in combating AD, highlighting the need for continued exploration for the future development of dietary supplements, nutraceuticals, or drugs, and their clinical application.

## Conclusion

Based on the results obtained, the aerial parts of *C. infortunatum* have the potential to serve as a safe, natural, and cost-effective herbal remedy for protecting against AD. Through LC-QTOF-MS/MS analysis, a tentative identification of 79 compounds from diverse classes was conducted, representing the first comprehensive metabolite profiling for this plant species. Notably, phenylpropanoids were the most abundant (19 metabolites), followed by phenolic acid and other phenolics (17), flavonoids (15), fatty acids (15), organic acids (9), and coumarins (4).

The oral administration of the extract was safe at a dose as high as 2000 mg/kg over 48 h, showing significant suppression of the in vitro AChE activity, with IC_50_ at 0.18 µg/mL (compared to 0.08 µg/mL for the standard drug DON). Furthermore, its anti-AD potential was investigated in SCOP-induced AD rats. The groups treated with 200 mg/kg and 400 mg/kg of the extract showed a significant effect, similar to that of DON. This was evident not only in the NOR test but also across various neurotransmitter and biochemical parameters, including ACh, AChE, DA, and NA, as well as anti-inflammatory mediators (TNF-α and IL-1β), antioxidant factors (GSH and MDA), and the AD marker Aβ protein. Histopathological investigation of the hippocampi confirmed previous findings, indicating that groups administered at these doses of the extract exhibited nearly normal hippocampal morphology compared to both the AD-induced group and the group administered 100 mg/kg of the extract.

These results point to a promising correlation between the major metabolites of the extract and its anti-AD activity. Further investigation is required, including in-depth mechanistic studies, extensive data collection, and the isolation of the most promising secondary metabolites or enriched fractions. Future research should also prioritize comprehensive ADMET analysis and bioavailability assessments to fully validate these compounds for clinical use, ensuring both efficacy and safety in drug development.

## Electronic supplementary material

Below is the link to the electronic supplementary material.

Supplementary Material 1

## Data Availability

Data is provided within the manuscript or supplementary information files.

## Abbreviations

ACh: Acetylcholine
AChE: Acetylcholinesterase
AD: Alzheimer’s disease
Aβ: Amyloid-β
Ag: Aglycon
C. infortunatum: Clerodendrum infortunatum
DA: Dopamine
DI: Discrimination index
DMSO: Dimethyl sulfoxide
DON: Donepezil
ELISA: Enzyme-linked immunosorbent assay
ESI: Electrospray ionization
GSH: Glutathione
IP: Intraperitoneal injection
IC: Inhibition concentration
IL-1β: Interleukin-1β
LC-QTOF-MS/MS: Liquid chromatography-quadrupole time-of-flight tandem mass spectrometry
LD: Lethal dose
MDA: Malondialdehyde
NA: Noradrenaline
NOR: Novel object recognition
R_t_: Retention time
ROS: Reactive oxygen species
SCOP: Scopolamine
SCOP-induced AD: Scopolamine-induced Alzheimer’s disease
TNF-α: Tumor necrosis factor-α

## Declarations

### ARRIVE guidelines

The study is reported in accordance with ARRIVE guidelines.

### A statement naming the person who identified the plant

The aerial parts of *C. infortunatum* were authenticated by Agriculture Engineer Therese Labib, a plant taxonomy consultant of the Ministry of Agriculture (Giza, Egypt).

### Voucher specimen statement

The plant material was collected with permission in compliance with national guidelines from the Agriculture Research Center, Giza, Egypt at “9 Cairo University Road, Giza District, Giza Governorate”. Samples of the plant material were deposited at the herbarium of the Faculty of Pharmacy, Cairo University, Cairo, Egypt (sample No.10.3.2021).
