# Supplementary material for: A comprehensive investigation of Clerodendrum Infortunatum Linn. using LC-QTOF-MS/MS metabolomics as a promising anti-alzheimer candidate
Source: Sci Rep. 2025 Jan 5;15:859. doi: 10.1038/s41598-024-82265-2 (PMC11701085; doi:10.1038/s41598-024-82265-2)
Supplement: Supplementary file 1 — Supplementary Material 1 [file 41598_2024_82265_MOESM1_ESM.docx]

**Supplementary figures**

**Fig. S1.** MS/MS spectrum of metabolite **4** (Table **1**)

**Fig. S2.** MS/MS spectrum of metabolite **8** (Table **1**)

**Fig. S3.** MS/MS spectrum of metabolite **9** (Table **1**)

**Fig. S4.** MS/MS spectrum of metabolite **15** (Table **1**)

**Fig. S5.** MS/MS spectrum of metabolite **26** (Table **1**)

**Fig. S6.** MS/MS spectrum of metabolite **27** (Table **1**)

**Fig. S7.** MS/MS spectrum of metabolite **29** (Table **1**)

**Fig. S8.** MS/MS spectrum of metabolite **32** (Table **1**)

**Fig. S9.** MS/MS spectrum of metabolite **36** (Table **1**)

**Fig. S10.** MS/MS spectrum of metabolite **37** (Table **1**)

**Fig. S11.** MS/MS spectrum of metabolite **48** (Table **1**)

**Fig. S12.** MS/MS spectrum of metabolite **49** (Table **1**)

**Fig. S13.** MS/MS spectrum of metabolite **57** (Table **1**)

**Fig. S14.** MS/MS spectrum of metabolite **34** (Table **1**)

**Fig. S15.** MS/MS spectrum of metabolite **39** (Table **1**)

**Fig. S16.** MS/MS spectrum of metabolite **46** (Table **1**)

**Fig. S17.** MS/MS spectrum of metabolite **53** (Table **1**)

**Fig. S18.** MS/MS spectrum of metabolite **58** (Table **1**)

**Fig. S19.** MS/MS spectrum of metabolite **47** (Table **1**)

**Fig. S20.** MS/MS spectrum of metabolite **54** (Table **1**)

**Fig. S21.** MS/MS spectrum of metabolite **55** (Table **1**)

**Fig. S22.** MS/MS spectrum of metabolite **60** (Table **1**)

**Fig. S23.** MS/MS spectrum of metabolite **51** (Table **1**)

**Fig. S24.** MS/MS spectrum of metabolite **59** (Table **1**)

**Fig. S25.** MS/MS spectrum of metabolite **62** (Table **1**)
